# Estimating the Unit Costs of Healthcare Service Delivery in India: Addressing Information Gaps for Price Setting and Health Technology Assessment

**DOI:** 10.1007/s40258-020-00566-9

**Published:** 2020-03-14

**Authors:** Pankaj Bahuguna, Lorna Guinness, Sameer Sharma, Akashdeep Singh Chauhan, Laura Downey, Shankar Prinja

**Affiliations:** 1 Department of Community Medicine and School of Public Health, Post Graduate Institute of Medical Education and Research, Chandigarh, 160012 India; 2 International Decision Support Initiative, London, UK; 3 School of Public Health, Imperial College London, London, W2 1NY UK

## Abstract

**Background:**

India’s flagship National Health insurance programme (AB-PMJAY) requires accurate cost information for evidence-based decision-making, strategic purchasing of health services and setting reimbursement rates. To address the challenge of limited health service cost data, this study used econometric methods to identify determinants of cost and estimate unit costs for each Indian state.

**Methods:**

Using data from 81 facilities in six states, models were developed for inpatient and outpatient services at primary and secondary level public health facilities. A best-fit unit cost function was identified using guided stepwise regression and combined with data on health service infrastructure and utilisation to predict state-level unit costs.

**Results:**

Health service utilisation had the greatest influence on unit cost, while number of beds, facility level and the state were also good predictors. For district hospitals, predicted cost per inpatient admission ranged from 1028 (313–3429) Indian Rupees (INR) to 4499 (1451–14,159) INR and cost per outpatient visit ranged from 91 (44–196) INR to 657 (339–1337) INR, across the states. For community healthcare centres and primary healthcare centres, cost per admission ranged from 412 (148–1151) INR to 3677 (1359–10,055) INR and cost per outpatient visit ranged from 96 (50–187) INR to 429 (217–844) INR.

**Conclusion:**

This is the first time cost estimates for inpatient admissions and outpatient visits for all states have been estimated using standardised data. The model demonstrates the usefulness of such an approach in the Indian context to help inform health technology assessment, budgeting and forecasting, as well as differential pricing, and could be applied to similar country contexts where cost data are limited.

**Electronic supplementary material:**

The online version of this article (10.1007/s40258-020-00566-9) contains supplementary material, which is available to authorized users.

## Key Points for Decision Makers

**Table:** 

There is an urgent need for healthcare cost data in India to inform priority setting, insurance reimbursement rates and budgeting.
A statistical cost function is used to estimate costs for settings where there currently are no data and provides a set of state-level unit costs.
The analysis shows the variability in healthcare costs across different settings and demonstrates the usefulness of such an approach in the absence of national cost datasets.

## Background

The costs of healthcare service delivery underpin many important policy decisions—from questions of affordability, to making choices between different technologies and innovations, to setting prices of health services. Many countries, and in particular, low- and middle-income countries, suffer from a lack of information in this area, creating an information vacuum that leads to opaque policy decisions and cost escalation in health services [1, 2]. As the modus operandi of public health systems involves the purchasing of services by the state using public money, and as international pressure to move towards systems of Universal Health Care (UHC) coverage grows, governments without cost information are increasingly vulnerable in their ability to engage in strategic purchasing of health services. Cost information allows governments to be better-equipped as price setters and allows for informed decisions around the allocation of resources between different healthcare services and technologies to ensure value for money.

The challenges arising from limited healthcare cost information are magnified in the highly complex and fragmented Indian health system, where public services are purchased and delivered by a mix of both private and state providers. India operates under a model of fiscal federalism, where health is primarily the responsibility of state governments. There are a multitude of state insurance schemes in operation across the country that have been introduced to tackle the inequitable access to healthcare [3]. At the central level, the first national government tax-funded scheme for the poor and vulnerable was initiated in 2008. In 2018, the national government then launched the Ayushman Bharat-Prime Minister’s Jan Arogya Yojana (AB-PMJAY), a tax-funded national insurance programme that aims to subsume earlier national and state schemes and to provide healthcare cover of 500,000 Indian Rupees (INR) for over 500 million poor beneficiaries [4]. Despite these different schemes, to date, state governments have not used systematic, evidence-based approaches to setting prices [5]. Until recently, the process of setting prices under AB-PMJAY and other insurance schemes has been somewhat haphazard, relying on surveys of insurance claims data and interviews with experts. The government explained the severity of the problem in 2018: “we don’t have costing studies in India” [6]. While the latter statement exaggerates the issue, there are limited studies, the majority of which focussed on a single disease, technology or site. Data on private expenditures are regularly collected by the National Sample Survey Office, but this is limited to patient expenditures on healthcare and cannot be broken down fully by disease or condition [7, 8]. Insurance claims data from 22 different government-funded schemes have also been collated into a single database; however, these estimates reflect prices agreed by tender and do not represent the cost of production [7, 9]. More recently, the availability of production cost data for the public sector has begun to grow with individual costing studies that have been carried out to explore the cost-effectiveness of different technologies as well as a series of primary costing studies [10–19]. However, the paucity of cost information remains a significant fact within the Indian health system [7, 20].

Compounding the general lack of cost information, a further challenge in the practical application of cost data for price setting and health technology assessment in India is the vast heterogeneity in costs of service delivery across different types of providers, levels of the system, states and geographical settings. Previously published cost studies have highlighted the variation in healthcare costs within the Indian health system [10–13, 20, 21]. This variation in unit costs arises as a result of supply factors due to different methods of production, ownership (public or private), differences in prices and wage rates as well as demand factors such as epidemiology, population density and socio-economic status of the local population [22–24]. Moreover, India’s size and federal governance structure with different modes of financing, delivery and purchasing of healthcare have given rise to the view that there is scope for ‘differential pricing’ across settings when setting reimbursement rates.

With sufficient data, it is possible to explore the determinants of cost using a statistical cost function and thereby enable the prediction of costs for different settings. The advantage of using a cost function approach is the ability to interpret the coefficients on key variables such as scale, geography and other factors and explore the impact of these factors that might drive cost variations [25–28]. At the global level, the World Health Organization (WHO) has used such an approach to predict country specific unit costs [29, 30]. In India, efforts, including those by the present authors, towards estimating costs and understanding the cost structures of healthcare facilities have begun [11–18, 21]. While these first studies have provided cost data for different levels of the health system, they have been undertaken in a limited number of facilities and states. Nonetheless, these data form the richest and most accurate source of cost data for publicly delivered health services in India to date and can be used to understand better what drives cost differences between facilities using statistical cost function analyses. Using these data and an average cost function approach, the aim of this paper is to develop models to identify the key drivers of unit costs of inpatient (IP) and outpatient (OP) services at primary and secondary level public health facilities in India for use in healthcare decision-making and to demonstrate the use of such a model in the prediction of unit costs for each Indian state.

## Methodology

### Development of the Models

The aims of the models are to predict unit costs of healthcare services for each state in India, at different service volumes by nature of service delivery (IP and OP care), and identify the degree to which other factors influence average costs. Unit costs are dependent on the total costs of input resources consumed (numerator) and output in terms of services provided (denominator). Total costs can broadly be divided into the ‘hotel’ components, i.e. human workforce, capital resources (like building and equipment) and overheads, and those associated with specific treatments, i.e. the costs of medicine, consumables and diagnostics, and are dependent on the beneficiaries. Hotel costs generally comprise 70–80% of hospital costs [13, 19, 31]. Other costs, i.e. those of medicines, consumables and diagnostics, are subject to different types of market influences than hotel type services, with patients typically contributing to a large share of these inputs in India [32]. These costs tend to be variable so that the average cost remains constant as volume of services changes. Hotel costs are more fixed in nature, with average costs varying with scale as well as other characteristics that might shape the production of a particular service. The cost function presented here is therefore designed to predict the hotel costs component of the average cost.

Our starting point is the WHO CHOICE refined model for predicting national-level unit costs (version 2) 2017/18 [30]. This model regresses unit costs against a set of explanatory variables such that:

$$\ln ({\text{UC}}) = \alpha_{0} + \alpha_{i} \mathop \sum \limits_{i = 1}^{n} \ln X_{i} + e_{i} \quad i = 1 \ldots n,$$

where UC is unit cost, $X_{i}$ are the explanatory variables, $\alpha_{0}$ and $\alpha_{1 \ldots n}$ are the estimated parameters, and *e* is the error term. Natural logs of both the unit cost and independent variables are used because of the inherently skewed nature of cost data. In addition, the equation can then be estimated using ordinary least squares methods, and the coefficients can be interpreted as elasticities [29].

An average cost function approach was chosen. In this approach, the unit cost is regressed against a set of independent variables, selected based on theory and previous empirical findings. Resource inputs and their prices are included to identify the influence of each, e.g. is it the price or quantity of doctors that explains more of the unit cost? Further, explanatory factors such as volume of services, geographical location, capacity utilisation, quality and the nature of the healthcare market can also be included [23, 25, 33–35]. As average cost is largely explained by volume of activity, the model needs to include output as an independent variable to isolate and identify the influence of each of the other variables. Careful attention then needs to be paid to potential multi-collinearity. Sensitivity analyses were run to verify the inclusion of output as an independent variable and the choice of average cost over total cost function.

Within a hospital the standard set of outputs are the OP visits, IP admissions or IP days. To estimate unit costs for a particular service within a large production unit, a regression model can be run separately for each different service based on the assumption that the production relationship between different services at any single facility is constant. In this case, separate models are needed for IP care and OP care.

### Data

Cost data are taken from the cost data surveys of 81 public sector facilities across six states in India: Punjab, Haryana, Tamil Nadu, Odisha, Himachal Pradesh and Kerala [11–13, 19]. The cost dataset comprised healthcare facility costs at district hospitals (DHs), community healthcare centres (CHC) and primary healthcare centres (PHC) (the sampling methods are described in Appendix 1; see the electronic supplementary material). Economic cost data were collected from a provider perspective using a mixed methodology. The first round of data collection took place in 2013; the second round took place in 2016. The costing methodology followed standard principles [36, 37], and the standardised methodology has been published elsewhere [11, 13, 19]. Costs from the first round were adjusted to 2015–2016 prices, in line with the second round, using a correction factor of 1.46 (source: https://www.calculatorstack.com/inflation-calculator-india.php).^1^ In line with service provision at the different health system levels, CHCs and DHs were included in the IP care model analysis. For the OP care model, the analysis included DHs, CHCs and PHCs.

Two adjustments were made to the cost dataset to address data issues. First, for the OP model estimation, 14 PHCs reported a value of zero for the number of beds. As number of beds was used as the measure of capital stock, and due to the need to take logs, we would have had to exclude these facilities from the OP model. To address this, we assumed the value 1 for the number of beds—as the lowest level of capital stock—for these facilities. Second, one CHC reported no admissions in the reference year and therefore was excluded from the IP analysis and the OP analysis runs where the admissions variable was required (models including capacity utilisation). No ethical clearance was required for this analysis of secondary data.

### Model Specification

A range of different variables were considered for inclusion in the models based on a combination of theoretical considerations and previous cost models (for example, see [22, 23, 30, 31, 38, 39]). To identify the best specification, we used a combination of guided stepwise linear regression informed by Table 1 and the perspective of a potential user of the model. Potential users attempting to generate a facility- or state-specific unit cost could include policy makers at the central or state level, academics and health technology assessment professionals. As data availability is limited in India, in particular, access to data on wages and human resources and even facility infrastructure variables, it was important to factor data accessibility into the decision around which variables to include in the final model. The variables considered included the outputs of each service category, prices and quantities of labour, proxy of capital and any other significant inputs into production, as well as supply-side factors that might lead to cost variation [22]. Importantly, the models needed to be constructed using readily accessible data to enable a user to predict costs for their settings.

**Table 1:** Proposed variables for input into the cost function

Model	WHO model [30]	Proposed: inpatient admission	Proposed: outpatient visit
Cost	Average cost	Cost per admission	Cost per outpatient visit
Technology (input mix)	GDP per capita	Labour input Capital stock	Labour input Capital stock
Price of labour		Price of labour	Price of labour
Price of capital		State healthcare expenditure per capita	State healthcare expenditure per capita
Capacity utilisation	Occupancy rate Average length of stay	Occupancy rate	Labour/output ratio
Case mix	Hospital level	Facility level	Facility level
Outputs	Inpatient admissions/outpatient visits	Inpatient admissions	Outpatient visits

*GDP* gross domestic product; *WHO* World Health Organization

Following Serje et al. four categories of labour should be considered [40]: professional, technical/auxiliary, clerks/secretaries and physical labourers. However, our data only allowed distinction between three categories: doctors, medical support staff (nurses/technicians/pharmacists) and other support staff. The capital stock can be represented by the number of beds at the facility for both IP and OP services. Number of beds gives an indication of the relative capital stock even where IP beds are equal to zero. Outputs used are hospitalisations for the IP model and OP visits for the OP model.

To account for the influence of supply-side factors, we included the level of health facility, capacity utilisation and state-level per capita health expenditure as well as two proxy variables to represent quality and the nature of the healthcare market. These proxy variables are the state health index, which reflects a combination of health outcomes, governance and inputs to the health sector [41], and the government “Aspirational District Programme” index, a composite index of socio-economic progress, health and education sector performance and basic infrastructure indicators [42]. The full set of variables considered for the models is listed and defined in Table 2. The best-fit models were then selected based on both statistical and theoretical considerations.

**Table 2:** List and definition of variables considered for each of the models

Category	Variable	Definition	Source
Cost	Unit cost	Total cost of inpatient services divided by number of hospitalisations; total cost of outpatient services divided by number of outpatient visits. All costs are presented in 2015–2016 INR	Cost dataset
	Total costs	Total costs are defined as the hotel costs and include human resources, equipment, building space, furniture and overheads. Treatment-specific costs of drugs, consumables, laboratory investigations are excluded	Cost dataset
Labour	Number of doctors	Number of medical doctors working at the health facility (full or part-time)	Cost dataset
	Number of technical staff	Combined number of nurses, pharmacists and other technical staff	Cost dataset
	Number of other staff	Combined number of non medical staff	Cost dataset
Capital	Number of beds	Number of inpatient beds at the health facility^a,b^	Cost dataset
Prices	Mean salary of doctors at facility	Mean wage of doctors working at the health facility	Cost dataset
	Mean salary of technical staff	Mean wage of technical staff (as defined above)	Cost dataset
	Mean salary of other staff	Mean wage of other staff (as defined above)	Cost dataset
Output	Hospitalisations per year	Number of inpatient admissions in 1 year for the facility	Cost dataset
	Annual outpatient consultations	Number of individual outpatient visits to the facility in 1 year	Cost dataset
Other characteristics	Dummy for health facility level	Health facility level as defined by the state in which the facility is located: DH, CHC and PHC	Cost dataset
	State health index	Baseline ranking of health sector incorporating aspects of health outcomes, governance and health sector inputs. A standardised measure available for all states^c^	[41]
	Aspirational District Index	Baseline ranking of districts according to a composite index of socio-economic, health and educator sector and infrastructure measures	[42]
	Capacity utilisation	Service outputs were adjusted for capacity utilisation using the following formulae: DH and CHC levels: bed occupancy rate = (number of patients × ALOS)/(number of beds × number of days)^d^ PHC: number of outpatient consultations/number of medical officers at the facility	Calculation based on cost data set
	State healthcare expenditure per capita	Public expenditure on health per capita at the state level	[43]

*ALOS* average length of stay, *DH* district hospital, *CHC* community healthcare centre, *INR* Indian Rupees, *PHC* primary healthcare centre

^a^Number of beds is a good reflection of the working capital of a facility. In addition, the variable is an easier variable to obtain for anyone wishing to use the model for further estimations

^b^14 PHCs did not report data on number of beds. As this was being used as a proxy for capital and because of the need to take logs, we assumed this value to be 1 where actual beds = 0

^c^To reflect the demand side, an additional variable capturing condition-specific data at the state or facility level would have been preferable, but standardised, reliable and good-quality data of this type are difficult to obtain for potential users of the model

^d^For one CHC, no routine inpatient admission happened in the reference year, so capacity utilisation for the inpatient model could not be calculated and this CHC was excluded from the analysis

### Model Selection

A best-fit model was then chosen for each of the OP and IP cost functions for the prediction of unit costs at DHs, CHCs and PHCs for all states in India. Base models for prediction were selected based on the trade-off between the data requirements and high predictive power of the model in terms of adjusted *R*-squared. First, models with multi-collinearity [variance influence factor (VIF) scores > 10] were excluded [44]. Subsequently models were listed in order of the adjusted *R*-squared. Starting with the model with the highest score, the models were assessed for their suitability to be used in the state-level predictions, according to data availability and ability to interpret the coefficient on the independent variable. The first model to fit these criteria was selected for the state-level cost predictions.

### Cost Predictions

The mean values and the 2.5% and 97.5% uncertainty limits for IP admissions and OP visits at DHs and CHCs were estimated for all states in India using the best-fit models. State-level data were identified for each of the variables using national-level data sources including the National Health Profile, the National Sample Survey Office (NSSO) “Health in India” report (71st round) and, where data were unavailable at the national level, state-specific health system websites [8, 45]. There were no centrally compiled data on hospitalisations and OP visits as reported by facilities. These state-level variables were therefore estimated from NSSO household survey data on hospitalisations and seeking medical care in the public sector combined with reported institutional deliveries and maternal and child healthcare visits. The summary data on hospitalisations and OP visits and the methodology for their estimation is provided in supplementary appendices 2 and 3 (see the electronic supplementary material).

The predicted unit cost generated from the models is a median value due to the log transformations. This was adjusted to the arithmetic mean by applying a smearing factor [30, 46]. Uncertainty intervals around the predicted values were also constructed by first generating a random sample of 1000 based on the mean and standard error of each of the coefficients in the model and extracting the values at the 2.5th and 97.5th percentiles. Versions of the models will further be made available online for individual users to estimate unit costs for their own settings (see https://www.healtheconomics.pgisph.in/costing_web/index.php).

### Model Validation

The cost prediction models were validated by using data and results from preliminary findings of an additional costing study funded by the Government of India^2^ [47]. Actual costs and the explanatory variables were extracted from the primary data collected from the sampled health facilities for this study. The explanatory variables were used to predict the facility unit cost estimates based on the state prediction models. The predicted cost estimates were then compared with the actual unit cost estimates generated as part of the national costing study. The study results are yet unpublished, so identifiers of the health facilities were kept confidential.

## Results

### Model Outcomes

Descriptive statistics for each model are reported in Table 3. The mean unit cost for an IP hospital admission across the sample was 2563 INR, and the mean number of hospitalisations per facility in a year was 10,467. The mean cost of an OP consultation was 113 INR, with an average of 76,129 consultations in a year. DHs represented 37.5% of the IP sample (DHs and CHCs only) and 19% of the OP sample (which also includes PHCs).

**Table 3:** Descriptive statistics of the cost data used in the estimation of the inpatient and outpatient cost models

A. Summary data for inpatient admissions at community healthcare centres and district hospitals												
Facility level	Cost per admission (INR)	Number of doctors	Number of other technical staff	Number of support staff	Mean monthly doctor’s salary (INR)	Mean monthly salary of other technical staff	Mean monthly salary of support staff	Number of beds	Number of admissions	Capacity utilisation	Per capita government healthcare expenditure in the state	State health index
District hospital												
Mean	3255.8	29.40	92.47	52.47	85,973.77	40,581.47	26,767.02	199.67	24,053.47	197.47	1183.53	60.80
*N*	15.0	15.00	15.00	15.00	15.00	15.00	15.00	15.00	15.00	15.00	15.00	15.00
Standard deviation	2974.8	12.32	72.41	29.41	24,343.38	8078.23	7313.70	131.31	32,761.39	253.68	455.42	13.19
Community healthcare centre												
Mean	2163.8	5.69	14.04	11.62	65,531.09	31,037.50	22,819.35	21.85	2629.58	72.32	1072.85	58.75
*N*	26.0	26.00	26.00	26.00	26.00	26.00	26.00	26.00	26.00	25.00	26.00	26.00
Standard deviation	2170.8	2.59	5.58	6.54	28,304.25	12,006.55	5255.27	12.24	2866.84	60.52	388.34	15.31
Total												
Mean	2563.3	14.37	42.73	26.56	73,010.12	34,529.19	24,263.62	86.90	10,467.59	119.25	1113.34	59.50
*N*	41.0	41.00	41.00	41.00	41.00	41.00	41.00	41.00	41.00	40.00	41.00	41.00
Standard deviation	2515.2	13.82	57.60	26.95	28,416.47	11,601.71	6299.90	116.82	22,134.66	170.64	412.02	14.44

B. Summary data for outpatient visits at primary healthcare centres, community healthcare centres and district hospitals												
Facility	Cost per outpatient visit (INR)	Number of doctors	Number of other technical staff	Number of support staff	Mean monthly doctor’s salary (INR)	Mean monthly salary of other technical staff (INR)	Mean monthly salary of support staff (INR)	Number of beds	Number of outpatient visits	Capacity utilisation	Per capita government healthcare expenditure in the state (INR)	State health index
District hospital												
Mean	174.0	29.4	92.5	52.5	85973.8	40581.5	26767.0	199.7	229891.2	197.5	1183.5	60.8
*N*	15.0	15.0	15.0	15.0	15.0	15.0	15.0	15.0	15.0	15.0	15.0	15.0
Standard deviation	88.7	12.3	72.4	29.4	24343.4	8078.2	7313.7	131.3	173935.7	253.7	455.4	13.2
Community healthcare centre												
Mean	106.2	5.7	14.0	11.6	65531.1	31037.5	22819.4	21.8	63246.7	72.3	1072.8	58.8
*N*	26.0	26.0	26.0	26.0	26.0	26.0	26.0	26.0	26.0	25.0	26.0	26.0
Standard deviation	56.1	2.6	5.6	6.5	28304.2	12006.6	5255.3	12.2	43082.2	60.5	388.3	15.3
Primary healthcare centre												
Mean	95.3	1.9	5.5	3.1	55582.3	28920.8	17555.1	3.4	26842.1	100.7	1182.5	60.4
*N*	40.0	40.0	40.0	38.0	40.0	40.0	38.0	40.0	40.0	40.0	40.0	40.0
Standard deviation	76.3	0.9	3.5	2.6	28573.6	8717.4	7159.5	3.7	21519.7	47.8	455.9	14.9
Total												
Mean	113.4	8.2	24.3	15.3	64403.8	31759.6	21036.7	45.7	76129.2	110.0	1147.5	59.9
*N*	81.0	81.0	81.0	79.0	81.0	81.0	79.0	81.0	81.0	80.0	81.0	81.0
Standard deviation	77.9	11.6	44.9	22.7	29641.8	10597.5	7486.4	92.7	108635.8	124.9	432.9	14.6

*INR* Indian Rupees

Table 4a and b present the results of the best-fit models for the IP and OP sample. The supplementary material reports on all the model runs that were carried out (Appendix 4; see the electronic supplementary material). The majority of the models had good predictive power with an adjusted R-squared greater than 0.7 both with and without inclusion of price and labour variables. The coefficients made theoretical and logical sense with relationships in the hypothesised direction and most were significant. Capacity utilisation was excluded from the reported models due to the presence of multi-collinearity. When output was excluded, the adjusted R-squared was found to be lower than when it was included, and a model run with total cost as the dependent variable yielded similar results to the average cost function (see Appendix 6). In the case of the IP cost estimation, the models that included information on quantity and price of labour performed better than those without—with adjusted R-squared scores all greater than 0.9. For the OP model, models that included the labour variables suffered from multi-collinearity. The models that included district ranking as a variable performed better than those that did not, but due to the difficulty of interpreting the coefficient on a ranking and the impracticality of using this for state-level estimates, these models were not considered for state-level estimations. The output variable has the greatest influence on the unit cost for both IP and OP care. Capital, in the form of number of beds, and the facility level were both important and significant predictors of unit cost. The state health index was significant for some models, but had a relatively smaller influence on the overall unit cost.

**Table 4:** Results of the unit cost function estimation

A. Inpatient model					
Variable	Model A	Model B	Model C^a^	Model D	Model E
Adjusted *R*^2^	0.936	0.878	0.778	0.777	0.709
Constant	2.415	5.896**	9.997**	12.301**	9.910**
Ln number of doctors	0.204**	0.445**			
Ln number of staff nurses/tech/pharmacists	0.323**				
Ln number of support staff	0.095				
Ln mean wages of doctors	0.251**	0.493**			
Ln mean wages of staff nurses/tech/pharmacists	0.713**				
Ln mean wages of support staff	− 0.168				
Ln number of beds	0.035	0.241**	0.402**	0.458**	0.725**
Ln annual hospitalisations	− 0.693**	− 0.753**	− 0.688**	− 0.722**	− 0.641**
Ln state health index			0.275		
Dummy DH	0.551**	0.335**	1.050**	1.035**	
Per capita state public health expenditure	–	–		− 0.161	

B. Outpatient model					
Variable	Model A	Model B	Model C^a^	Model D	Model E
Adjusted *R*^2^	0.823	0.631	0.580	0.536	0.7616
Constant	2.302**	9.295**	7.556**	7.152**	5.418
Ln number of doctors					0.489
Ln number of staff nurses/tech/pharmacists	0.228**				
Ln number of support staff	0.208**				
Ln mean wages of doctors	0.483**				0.472
Ln mean wages of staff nurses/tech/pharmacists	0.456**				
Ln mean wages of support staff	0.046				
Ln number of beds	0.061	0.270**	0.294**	0.364**	0.113
Ln annual OP consultations	− 0.754**	− 0.523**	− 0.536**	− 0.484**	− 0.76
Ln state health index	− 0.333*	0.576*	0.464**	0.415*	0.248
District ranking		− 0.018**			
Ln capacity utilisation		− 0.056			
Per capita state public health expenditure		− 0.265			
Dummy DH	0.369**	0.619**	0.574**		0.268

*DH* district hospital, *OP* outpatient

**Significant at 95%; significant at 90%

^a^Model selected for state-level predictions

### Cost Predictions

**Fig. 1:**
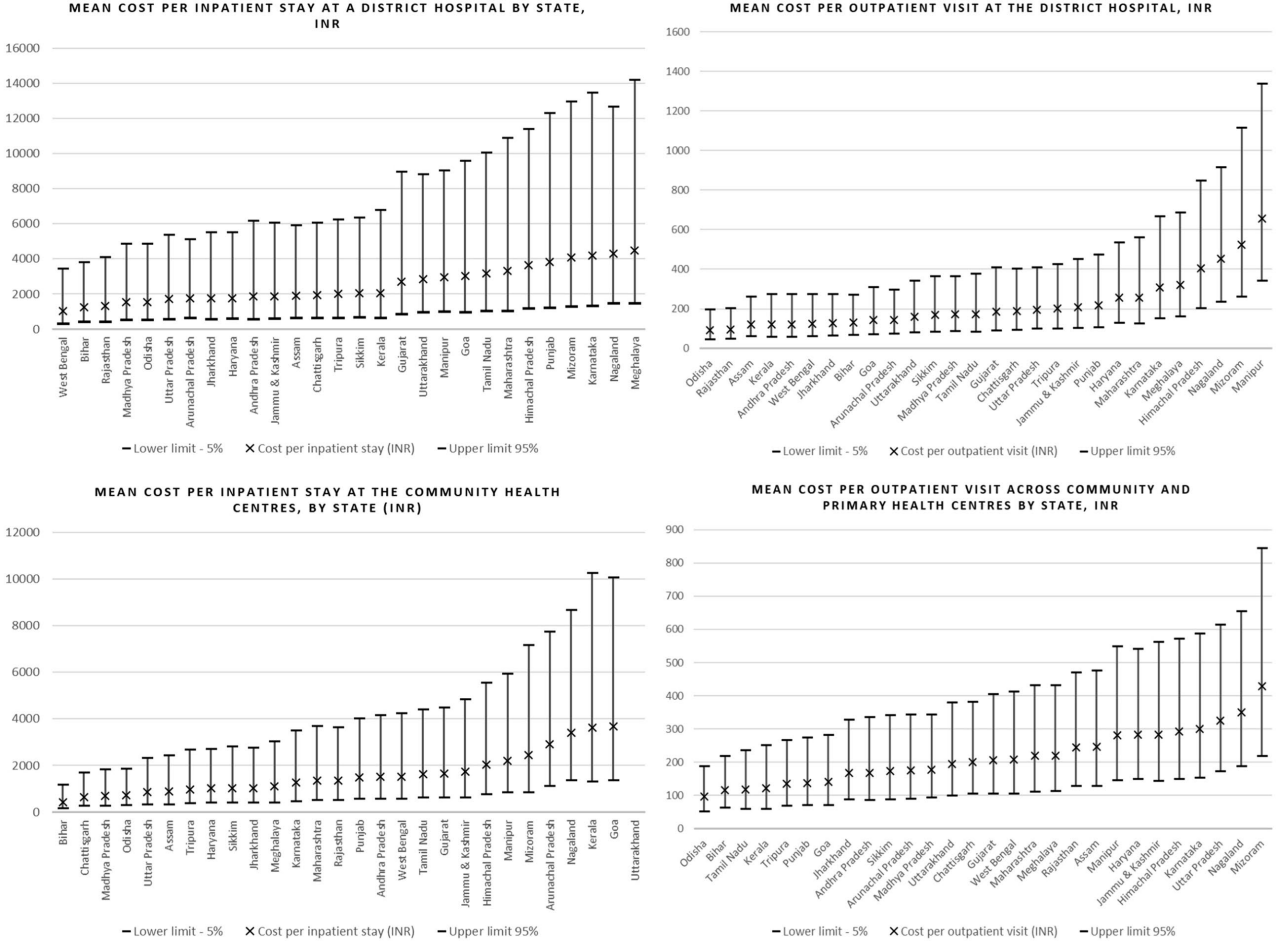
State-level inpatient admission and outpatient visit unit cost predictions, Indian Rupees (INR)

Model C (see Table 4a and b) was used to predict the state-level unit costs. Model C was chosen as it fits both the criterion of access to data on each variable at the health facility or state/district health departments and statistically had a good R-squared value. The state-level estimates for IP admissions and OP visits at DHs and CHCs are shown in Fig. 1 and Appendix 5 (see the electronic supplementary material, supplementary tables A5.1–A5.4). The mean cost of an IP admission at a DH was 2502 INR, ranging from 1028 (313–3429) INR in West Bengal to 4499 (1451–14,159) INR in Meghalaya. Inpatient admissions at the CHC level had a mean cost of 1601 INR and ranged from 412 (148–1151) INR in Bihar to 3677 (1359–10,055) INR in Goa. There is a wide range in the uncertainty intervals. For OP care, at the DH level, the mean cost of an OP visit was 224 INR, ranging from 91 (44–196) INR in Odisha to 657 (339–1337) INR in Manipur. The mean OP visits cost across CHCs and PHCs was 214 INR, ranging from 96 (50–187) INR in Odisha to 429 (217–844) INR in Mizoram.

In the validation exercise, the actual unit costs from the sampled facilities all fall within the uncertainty intervals for the unit costs predicted by the models. The difference between the actual and predicted costs ranged from − 2 to 62% in the IP model and − 90% to 30% for the OP model (see Table 5).

**Table 5:** Results of the validation exercise: actual versus predicted unit costs for eight facilities

Facility	Inpatient unit cost (INR)					Outpatient unit cost (INR)				
	Predicted	Lower limit	Upper limit	Actual	% difference (actual − predicted)	Predicted	Lower limit	Upper limit	Actual	% difference (actual − predicted)
A	842	269	2672	828	− 2	149	74	317	78	− 90
B	793	256	2487	2104	62	125	62	264	178	30
C	1137	372	3518	1717	34	166	83	348	125	− 33
D	2268	694	7522	2082	− 9	179	85	396	114	− 57
E	1254	416	3833	1761	29	227	115	471	184	− 24
F	1102	372	3310	2174	49	201	103	412	127	− 58
G	1188	389	3675	1406	16	195	98	407	110	− 76
H	1896	624	5849	2690	30	110	54	235	103	− 8

*INR* Indian Rupees

## Discussion

This paper has described the development of a set of models to predict the mean hotel costs of IP admissions and OP visits in Indian healthcare facilities. The models have been used to generate state-level estimates of the cost of IP admissions and OP visits in India. This is the first time cost estimates for IP admissions and OP visits across all the states have been estimated based on standardised data, and no other standardised data on economic costs exist in this form. The models and the cost estimates are a valuable resource for health policy-makers and planners, and can also be utilised to inform important policy-relevant research. In the absence of healthcare cost information in India, these models can inform multiple high-level policy decisions regarding the design and shape of health benefits packages and insurance schemes, including price setting, health technology assessment, budgeting and forecasting. They provide a method with which to estimate facility-level unit costs for both IP and OP care without the need to undertake a full costing exercise, saving both time and money, and avoiding the need to use an inaccurate national average.

The variation in cost estimates across the states, predicted by the models, confirms the need for state-level information in estimating costs. But the differences in unit costs can be driven by a number of factors, including different levels of service quality and input mix, case mix, price and wage levels and/or the nature of the referral systems [23]. Due to the nature of the models developed here, hospitalisation or OP visit rate have the strongest influence on the unit cost. As a result, unit costs in the states where the DHs have a higher patient load generally have lower costs. In contrast, the states with low population density (such as Nagaland and Mizoram) consistently have higher unit costs across the different sets of predictions. It is also notable that the states that score highest on the state health index (Mizoram, Kerala, Punjab and Tamil Nadu) all have IP unit costs that fall in the higher cost half of states. Those states that score lower on the state health index (Odisha, Rajasthan, Bihar and Nagaland) tend to have lower unit costs.

As well as a wide range in the unit costs across the states, there is some uncertainty in the point estimates seen in the uncertainty intervals. Some of this uncertainty is driven by the choice of model for the state-level predictions. Facility-level unit cost predictions are likely to need a better specified model that captures individual facility characteristics, including management, quality of care, wage rates and labour time. The model runs presented in Appendix 4 (see electronic supplementary material) confirm that state-level models could be improved. However, for this analysis, it was not possible to use models with the highest adjusted *R*-squared score (e.g. model A in Table 4a and b), as quality data on the average price of labour and the average quantity of human resources at facilities in each state were not available. In addition, the models have been developed using data from only six states and include only public facilities. Cost data from more states would help strengthen and further validate the model predictions for each state. The government funded costing study currently underway [47] has potential to expand the data set and include the private sectors and tertiary providers in an increased number of states.

Further to these limitations, the critical variables of hospital admissions and OP visits were estimated using household reported data. Ideally, facility-reported admissions and visit data from the Health Management Information System (HMIS) would have been used, but these were not accessible at the central level. There is also state variation in reporting and quality of data on infrastructure. In addition, differences in patient characteristics would further strengthen the estimates, but these are not consistently available either. To refine the modelling approach, improving the accessibility and quality of HMIS data is vital. To address this uncertainty, a validation procedure was carried out in which model predictions were compared with actual unit costs for individual sites. While there were some differences between the actual and predicted costs, the actual unit costs for the selected facilities fall within the upper and lower bounds of the predicted costs for the same sites. This suggests that the results from the models are relatively robust, but that larger samples of cost data are needed to narrow the uncertainty intervals. More and better data on health facility costs, state-level health system infrastructure and service provision, in particular, facility-level average admission and OP visit rates, will increase the predictive power of the models.

The variation in costs across the states suggests that costs have the potential to be more closely aligned to each other. In other words, there is scope for improving efficiency and allocation of resources. Ideally unit costs would be aligned around those states that are more efficient at producing quality healthcare services that are equitably distributed, and therefore these estimates need to be used with caution. However, the level of efficiency with respect to health outcomes or quality health services of these facilities is currently unknown, suggesting further research to identify the most efficient states or facilities would be highly beneficial. The state-level estimates also provide a way to assist in the prediction of costs of healthcare interventions or treatment of conditions and can assist with setting ‘differential prices’ for insurance reimbursement. By characterising the relationship between healthcare costs between different states, it is possible to use this relationship to predict costs or set prices for specific interventions. For example, as the model predicts that DH IP costs are 1.5 times higher in Kerala than Odisha; then if cost data are available for Kerala, one approach would be to assume that an IP procedure such as a caesarean section will be 1.5 times the cost of a caesarean section in Odisha. Again, further research is required to validate this as a robust method for setting differential prices for treatment of individual conditions.

In using statistical methods to predict costs, the analysis confirms that unit costs in India are not explained by scale of activity (admissions/OP visits) and capacity (number of beds) alone and that a modelling approach such as this is a better method to obtain state-level average costs estimates. While limitations do persist and therefore the results and, in particular, point estimates need to be used with caution, this first attempt at modelling facility costs in India represents a step forward in providing a more evidence-based approach to cost estimation.

## Conclusion

There exists a dearth of cost information within India. We describe here a novel means of estimating cost functions within the Indian healthcare system, which can provide a robust means of filing information gaps in the costing evidence base. An adaptation of the WHO method for estimating unit costs was applied at a country level to generate unit costs estimates for different states in India. The models estimated were statistically robust and found significant variation in unit costs between the states and levels of the health system. Further cost and health system data are still needed to improve the cost evidence base in India. Despite this, the cost function approach is a useful tool for the Indian context, and can be further developed as the evidence base expands at the district level and below, as well as for tertiary level and private sector facilities.

## Electronic Supplementary Material

Below is the link to the electronic supplementary material.

Supplementary material 1 (DOCX 118 kb)

## Data Availability

The data used for the analysis can be found on the website of the National Health System Cost Database for India at https://www.healtheconomics.pgisph.in/costing_web/index.php. The models tested in the analysis are documented and included in the electronic supplementary material (Appendix 4).

## Footnotes

1 Costs can be converted to current prices using the converter at https://www.healtheconomics.pgisph.in/costing_web/index.php.

2 The additional costing is being carried out in 11 out of 28 Indian states. The study is ongoing, with no planned release date at the time of the analysis presented here. While the new study moves the country closer to comprehensive cost information, there will still be considerable gaps, warranting the continued exploration of the cost function approach to estimating costs as a complement to the primary data.
