# Supplementary material for: Estimating the Unit Costs of Healthcare Service Delivery in India: Addressing Information Gaps for Price Setting and Health Technology Assessment
Source: Appl Health Econ Health Policy. 2020 Mar 14;18(5):699–711. doi: 10.1007/s40258-020-00566-9 (PMC7519005; doi:10.1007/s40258-020-00566-9)
Supplement: Supplementary file 1 — Supplementary material 1 (DOCX 118 kb) [file 40258_2020_566_MOESM1_ESM.docx]

# Appendix 1: Sampling methodology for the cost data

The present study used the cost data collected in six diverse states of India namely Himachal Pradesh (HP), Tamil Nadu (TN), Kerala, Odisha, Punjab and Haryana, collected in two rounds.

The first round of data collection was undertaken in 2013 covering Punjab, Haryana and Himachal Pradesh - three northern states of India. A multistage stratified random sampling was employed to select the districts and health facilities. Firstly, 10% of districts were chosen randomly from each state. Secondly, within each selected district, one community health center (CHC) and 10% of primary health centers (PHC) were selected by simple random sampling.^^[[1]](#footnote-1)^^

The second round of data collection took place in 2016. The geographic spread was broader in the second round, covering Kerala, TN, Odisha and HP states of India. HP represented a hilly state in North India with distinct characteristics from the other states like population norms, government spending, availability of healthcare infrastructure and utilisation rates and hence was covered in both the rounds. The states of Kerala and TN represented the southern region and are characterised by the best functioning models of health systems within the country. In contrast, Odisha represented a state with a poorly functioning health system. In the first stage of sampling, all the districts in each state were divided into three strata using the district ranking of health services according to socio-economic and demographic characteristics, compiled by the International Institute of Population Sciences (IIPS). In the case of TN and Odisha, however, the overall districts were divided into 2 strata. A district from each strata of each state was selected based on simple random sampling. In the second stage, 15% of the CHCs in each of the selected districts were selected randomly. A total of 10 DHs and 19 CHCs were selected across the four states.^^[[2]](#footnote-2)^^ More details pertaining to methods and sampling of costing surveys are provided in table S1.

The methods are published in the following papers:

Prinja S, Gupta A, Verma R, Bahuguna P, Kumar D, Kaur M, et al. (2016) Cost of Delivering Health Care Services in Public Sector Primary and Community Health Centres in North India. PLoS ONE 11(8): e0160986. doi:10.1371/journal.pone.0160986

Prinja S, Chauhan AS, Bahuguna P, Selvaraj S, Muraleedharan VR, Sundararaman T. Cost of Delivering Secondary Healthcare Through the Public Sector in India. PharmacoEconomics - Open. Springer International Publishing; 2019;1–13

Prinja S, Balasubramanian D, Jeet G, Verma R, Kumar D, Bahuguna P, et al. Cost of delivering secondary-level health care services through public sector district hospitals in India. Indian J Med Res. 2017;12.

Corresponding author for all papers is:

Dr Shanka Prinja, School of Public Health, Post Graduate Institute of Medical Education and Research, Chandigarh, India

email: shankarprinja@gmail.com

**Table S1: Details of two phases of costing surveys**

| **Parameter** | **Phase 1** | **Phase 2** |
| --- | --- | --- |
| Year of data collection | 2013 | 2016 |
| States | - Himachal Pradesh - Punjab - Haryana | - Himachal Pradesh - Tamil Nadu - Kerala - Odisha |
| Sampling Methodology | Multistage stratified random sampling | Multistage stratified random sampling |
| - District Selection | - 10% Districts chosen randomly from each state | - The districts in each state were divided into two (Odisha and TN) or three strata (HP and Kerala) based on socioeconomic and demographic characteristics (IIPS rankings) - Random selection of one district from each strata for each state |
| - Facility Selection | - 1 District hospital from each district - 1 Community health centre from each district - 10% of Primary health centres from each district | - 1 District hospital from each district - 15% of Community health centres from each selected district |
| Study Sample | Himachal Pradesh   - District – 1 - Facilities - 18 (PHC – 2, SC – 16)   Haryana   - District – 4 - Facilities – 31 (DH – 3, CHC – 4, PHC – 3, SC – 21)   Punjab   - District – 4 - Facilities – 19 (DH – 2, CHC – 3, PHC – 2, SC – 12) | Himachal Pradesh   - District – 4 - Facilities – 18 (DH – 3, CHC – 3, PHC – 6, SC – 6)   Odisha   - District – 6 - Facilities – 35 (DH – 2, CHC – 7, PHC – 10, SC – 16)   Kerala   - District – 6 - Facilities – 38 (DH – 3, CHC – 6, PHC – 10, SC – 19)   Tamil Nadu   - District – 4 - Facilities – 22 (DH – 2, CHC – 3, PHC – 7, SC – 10) |

Appendix 2: Summary statistics used in the state level mean unit cost estimation

| *PHC beds assumed to be zero; ^ Source: State Health Index Report; ^^Source: Annual Health Profile 2018 - except for Sikkim and Goa (and W. Bengal for DH) where estimate is from state level HMIS; **Source: NSSO 2014 and Annual Health Profile 2018 |
| --- |

Appendix 3: Estimating of inpatient admissions and outpatient visits by state

Mathematical formulations for the estimation of inpatient admissions and outpatient visits

State-wise estimation of number of hospitalizations per district hospital

h_ω_ = ((R_r_ * R_P_) + (U_r_ * U_P_)) * p_ω_

h_ω_ = State-specific annual number of hospitalisations in public sector hospitals

R_r_ = State-specific rural annual hospitalization rate (per thousand)

R_P_ = State-specific rural population

U_r_ = State-specific urban annual hospitalization rate (per thousand)

U_P_ = State-specific urban population

p_ω_= State-specific proportional share of public sector hospitalisations

ω = 1(State A), 2(State B) ……… (State N)

^ω^h_DH_ = (h_ω_ * (^ω^B_DH_/ (^ω^B_DH_ + ^ω^B_MC_)))/ ^ω^N_DH_ ……………………… (1)

^ω^h_DH_ = State-specific annual number of hospitalisations per district hospital

h_ω_ = State-specific annual number of hospitalisations in public sector hospitals

^ω^B_DH_ = State-specific total number of beds in public sector district hospitals

^ω^B_MC_ = State-specific total number of beds in public sector medical colleges

^ω^N_DH_ = State-specific total number of district hospitals

ω = 1(State A), 2(State B) ……… (State N)

State-wise estimation of number institutional deliveries per district hospital

ID_ω_ = (((R_BR_ * R_P_) + (U_BR_ * U_P_)) + ((R_SBR_ * R_P_) + (U_SBR_ * U_P_))) * ^ω^p_ID_

ID_ω_ = State-specific annual number of institutional deliveries in public sector hospitals

R_BR_ = State-specific rural birth rate (per thousand)

R_P_ = State-specific rural population

U_BR_ = State-specific urban birth rate (per thousand)

U_P_ = State-specific urban population

R_SBR_ = State-specific rural stillbirth rate (per thousand)

U_SBR_ = State-specific urban stillbirth rate (per thousand)

^ω^p_ID_ = State-specific proportional share of public sector institutional deliveries

ω = 1(State A), 2(State B) ……… (State N)

^ω^ID_DH_ = (ID_ω_ * (^ω^B_DH_/ (^ω^B_DH_ + ^ω^B_MC_)))/ ^ω^N_DH_ …………………… (2)

^ω^ID_DH_ = State-specific annual number of institutional deliveries per district hospital

ID_ω_ = State-specific annual number of institutional deliveries in public sector hospitals

^ω^B_DH_ = State-specific total number of beds in public sector district hospitals

^ω^B_MC_ = State-specific total number of beds in public sector medical colleges

^ω^N_DH_ = State-specific total number of district hospitals

ω = 1(State A), 2(State B) ……… (State N)

Adding (1) and (2), we get

^ω^H_DH_ = ^ω^h_DH_ + ^ω^ID_DH_

^ω^H_DH_ = State-specific annual number of hospitalisations (including institutional deliveries) per district hospital

^ω^h_DH_ = State-specific annual number of hospitalisations per district hospital

^ω^ID_DH_ = State-specific annual number of institutional deliveries per district hospital

ω = 1(State A), 2(State B) ……… (State N)

State-wise estimation of number of hospitalizations per non-district hospital

^ω^h_NDH_ = (((R_r_ * R_P_) + (U_r_ * U_P_)) * p_ω_ )/ ^ω^N_NDH_ ……………………… (3)

^ω^h_NDH_ = State-specific annual number of hospitalisations per public sector non-district hospitals

R_r_ = State-specific rural annual hospitalization rate (per thousand)

R_P_ = State-specific rural population

U_r_ = State-specific urban annual hospitalization rate (per thousand)

U_P_ = State-specific urban population

p_ω_= State-specific proportional share of hospitalisations for public sector non-district hospitals

^ω^N_NDH_ = State-specific total number of public sector non-district hospitals

ω = 1(State A), 2(State B) ……… (State N)

State-wise estimation of number of institutional deliveries per non-district hospital

^ω^ID_NDH_ = (((R_BR_ * R_P_) + (R_SBR_ * R_P_)) * ^ω^p_ID_ )/^ω^N_NDH_ …………………. (4)

^ω^ID_NDH_ = State-specific annual number of institutional deliveries per public sector non-district hospitals (rural)

R_BR_ = State-specific rural birth rate (per thousand)

R_P_ = State-specific rural population

R_SBR_ = State-specific rural stillbirth rate (per thousand)

^ω^p_ID_ = State-specific proportional share of institutional deliveries for public sector non-district hospitals

^ω^N_NDH_ = State-specific total number of public sector non-district hospitals (rural)

ω = 1(State A), 2(State B) ……… (State N)

Adding (3) and (4), we get

^ω^H_NDH_ = ^ω^h_NDH_ + ^ω^ID_NDH_

^ω^H_NDH_ = State-specific annual number of hospitalisations (including institutional deliveries) per non-district hospital

^ω^h_NDH_ = State-specific annual number of hospitalisations per public sector non-district hospitals

^ω^ID_NDH_ = State-specific annual number of institutional deliveries per public sector non-district hospitals (rural)

ω = 1(State A), 2(State B) ……… (State N)

State-wise estimation of number of outpatient consultations per district hospital

^ω^OP_NDH_ = ((((R_θ_ * R_P_) + (U_θ_ * U_P_))* 365/(15*1000)) * p_ω_ )/ ^ω^N_NDH_ ………… (5)

^ω^OP_NDH_ = State-specific annual number of out-patient visits per public sector district hospital

R_θ_ = State-specific rural illness rate in last 15 days (per thousand)

R_P_ = State-specific rural population

U_θ_ = State-specific urban illness rate in last 15 days (per thousand)

U_P_ = State-specific urban population

p_ω_= State-specific proportional urban share of public sector out-patient visits

^ω^N_NDH_ = State-specific total number of district hospitals

ω = 1(State A), 2(State B) ……… (State N)

State-wise estimation of number of antenatal visits per -district hospital

^ω^ANC_DH_ = ((^ω^ANC_N_ * 3) * ^ω^p_ANC_ ) / ^ω^N_DH_ ………………………… (6)

^ω^ANC_DH_ = State-specific annual number of antenatal visits per district hospital

^ω^ANC_N_ = State-specific number of women receiving 3 antenatal care (visits) in public sector hospitals

^ω^p_ANC_ = State-specific proportional share of antenatal visits in public sector district hospitals

^ω^N_DH_ = State-specific total number of district hospitals

ω = 1(State A), 2(State B) ……… (State N)

Adding (5) and (6), we get

^ω^OPV_DH_ = ^ω^OP_DH_ + ^ω^ANC_DH_

^ω^OPV_DH_ = State-specific annual number of out-patient visits (including antenatal visits) per district hospital

^ω^OP_DH_ = State-specific annual number of out-patient visits per district hospital

^ω^ANC_DH_ = State-specific annual number of antenatal visits per district hospital

ω = 1(State A), 2(State B) ……… (State N)

State-wise estimation of number of outpatient consultations per non-district hospital

^ω^OP_DH_ = ((((R_θ_ * R_P_) + (U_θ_ * U_P_))* 365/(15*1000)) * p_ω_ )/ ^ω^N_NDH_ ……… (7)

^ω^OP_NDH_ = State-specific annual number of out-patient visits per public sector non-district hospital

R_θ_ = State-specific rural illness rate in last 15 days (per thousand)

R_P_ = State-specific rural population

U_θ_ = State-specific urban illness rate in last 15 days (per thousand)

U_P_ = State-specific urban population

p_ω_= State-specific proportional rural share of public sector out-patient visits

ω = 1(State A), 2(State B) ……… (State N)

State-wise estimation of number of antenatal visits per non-district hospital

^ω^ANC_NDH_ = ((^ω^ANC_N_ * 3) * ^ω^p_ANC_ ) / ^ω^N_NDH_ ……………………… (8)

^ω^ANC_NDH_ = State-specific annual number of antenatal visits per district hospital

^ω^ANC_N_ = State-specific number of women receiving 3 antenatal care (visits) in public sector hospitals

^ω^p_ANC_ = State-specific proportional share of antenatal visits in public sector rural hospitals

^ω^N_NDH_ = State-specific total number of non-district hospitals

ω = 1(State A), 2(State B) ……… (State N)

Adding (7) and (8), we get

^ω^OPV_NDH_ = ^ω^OP_NDH_ + ^ω^ANC_NDH_

^ω^OPV_NDH_ = State-specific annual number of out-patient visits (including antenatal visits) per non-district hospital

^ω^OP_NDH_ = State-specific annual number of out-patient visits per non-district hospital

^ω^ANC_NDH_ = State-specific annual number of antenatal visits per non-district hospital

ω = 1(State A), 2(State B) ……… (State N)

Appendix 4: Results of the model runs for inpatient and outpatients.

A 4.1 Hospitalisations model runs and selection

A 4.1 Outpatient visits model runs and selection

Appendix 5: State level unit costs

Table A5.1: Inpatient unit costs by state – District Hospital

Table A5.2: Inpatient unit costs by state – CHC and PHC

Table A5.3: Outpatient unit costs by state – District Hospital

Table A5.4: Outpatient unit costs by state – CHC and PHC

# Appendix 6 – Sensitivity Analysis

Percentage difference between the unit cost estimate derived from the total cost function and the unit cost estimate derived from the base case unit cost function

| **State** | **OP non DH** | **OP DH** | **IP non DH** | **IP DH** |
| --- | --- | --- | --- | --- |
| Andhra Pradesh | 1.90% | 1.74% | 2.55% | 2.52% |
| Arunachal Pradesh | 1.90% | 1.91% | 2.52% | 2.61% |
| Assam | 1.90% | 1.79% | 2.56% | 2.54% |
| Bihar | 2.02% | 1.84% | 2.58% | 2.58% |
| Chattisgarh | 1.99% | 1.82% | 2.63% | 2.55% |
| Goa | 1.86% | 1.79% | 2.49% | 2.52% |
| Gujarat | 1.93% | 1.76% | 2.56% | 2.50% |
| Haryana | 1.95% | 1.83% | 2.58% | 2.55% |
| Himachal Pradesh | 1.92% | 1.85% | 2.54% | 2.53% |
| Jammu & Kashmir | 1.89% | 1.80% | 2.53% | 2.54% |
| Jharkhand | 1.93% | 1.80% | 2.58% | 2.55% |
| Karnataka | 1.91% | 1.79% | 2.55% | 2.49% |
| Kerala | 1.86% | 1.78% | 2.50% | 2.54% |
| Madhya Pradesh | 1.92% | 1.80% | 2.59% | 2.54% |
| Maharashtra | 1.92% | 1.77% | 2.56% | 2.50% |
| Manipur | 1.91% | 1.87% | 2.53% | 2.54% |
| Meghalaya | 1.93% | 1.81% | 2.57% | 2.50% |
| Mizoram | 1.92% | 1.84% | 2.50% | 2.52% |
| Nagaland | 1.93% | 1.84% | 2.51% | 2.51% |
| Odisha | 1.93% | 1.77% | 2.60% | 2.54% |
| Punjab | 1.94% | 1.80% | 2.58% | 2.51% |
| Rajasthan | 1.89% | 1.77% | 2.53% | 2.54% |
| Sikkim | 1.92% | 1.83% | 2.58% | 2.55% |
| Tamil Nadu | 1.93% | 1.81% | 2.57% | 2.53% |
| Tripura | 1.88% | 1.80% | 2.56% | 2.53% |
| Uttar Pradesh | 1.90% | 1.78% | 2.56% | 2.52% |
| Uttarakhand | 1.88% | 1.81% | - | 2.52% |
| West Bengal | 1.89% | 1.75% | 2.54% | 2.54% |
| Average | 1.92% | 1.81% | 2.55% | 2.53% |

1. Prinja S, Gupta A, Verma R, Bahuguna P, Kumar D, Kaur M, et al. (2016) Cost of Delivering Health Care Services in Public Sector Primary and Community Health Centres in North India. PLoS ONE 11(8): e0160986. doi:10.1371/journal.pone.0160986 [↑](#footnote-ref-1)
2. Prinja S, Chauhan AS, Bahuguna P, Selvaraj S, Muraleedharan VR, Sundararaman T. Cost of Delivering Secondary Healthcare Through the Public Sector in India. PharmacoEconomics-open. 2019 Aug 29:1-3. [↑](#footnote-ref-2)
